# Supplementary figures and images for: Selection of trait-specific markers and multi-environment models improve genomic predictive ability in rice
Source: PLoS One. 2019 May 6;14(5):e0208871. doi: 10.1371/journal.pone.0208871 (PMC6502484; doi:10.1371/journal.pone.0208871)

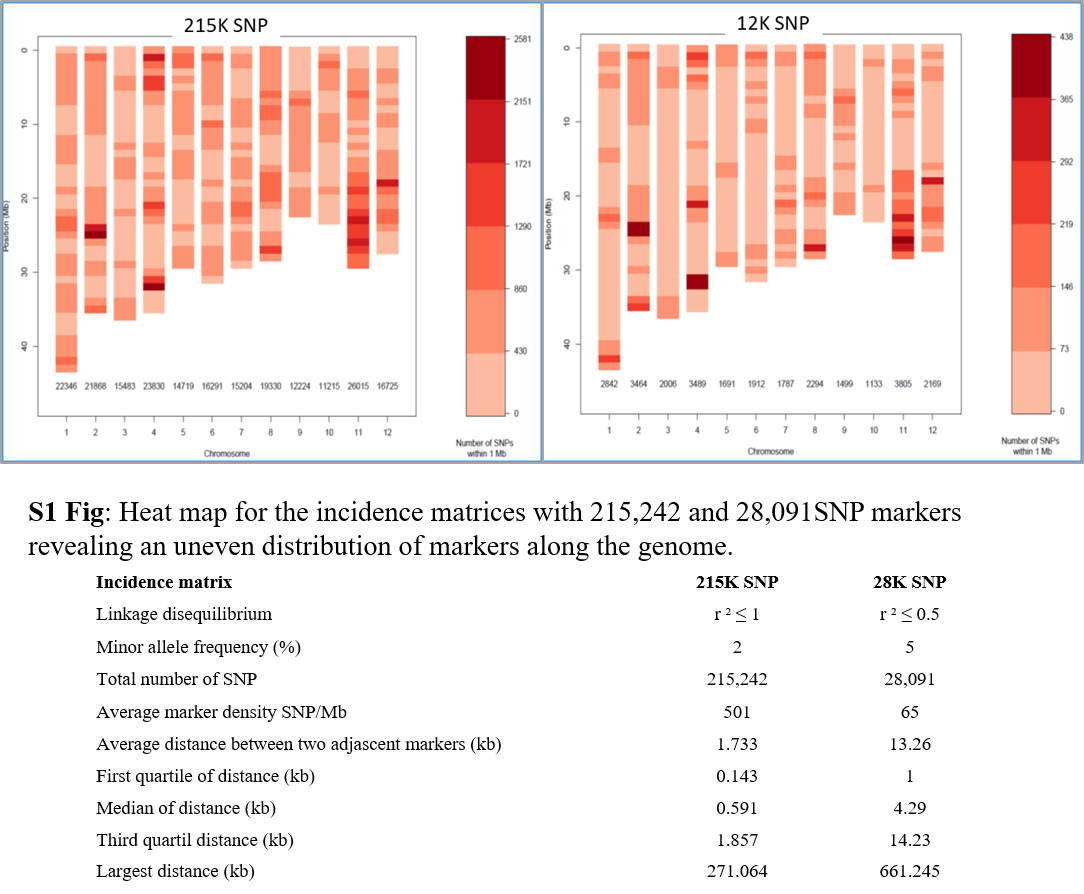

Supplement: S1 Fig — (TIF) [file pone.0208871.s007.tif]

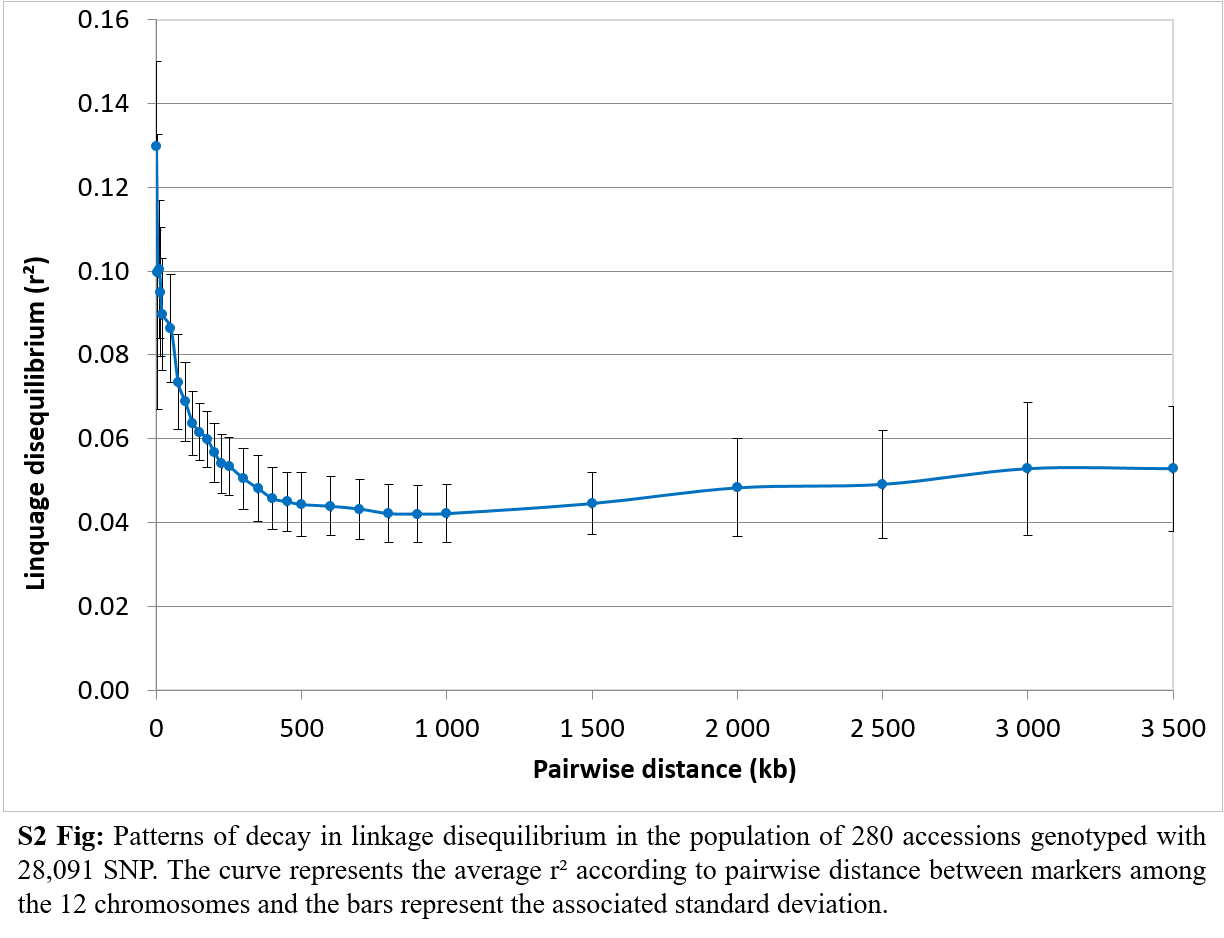

Supplement: S2 Fig — The curve represents the average r² according to pairwise distance between markers among the 12 chromosomes and the bars represent the associated standard deviation. (TIF) [file pone.0208871.s008.tif]

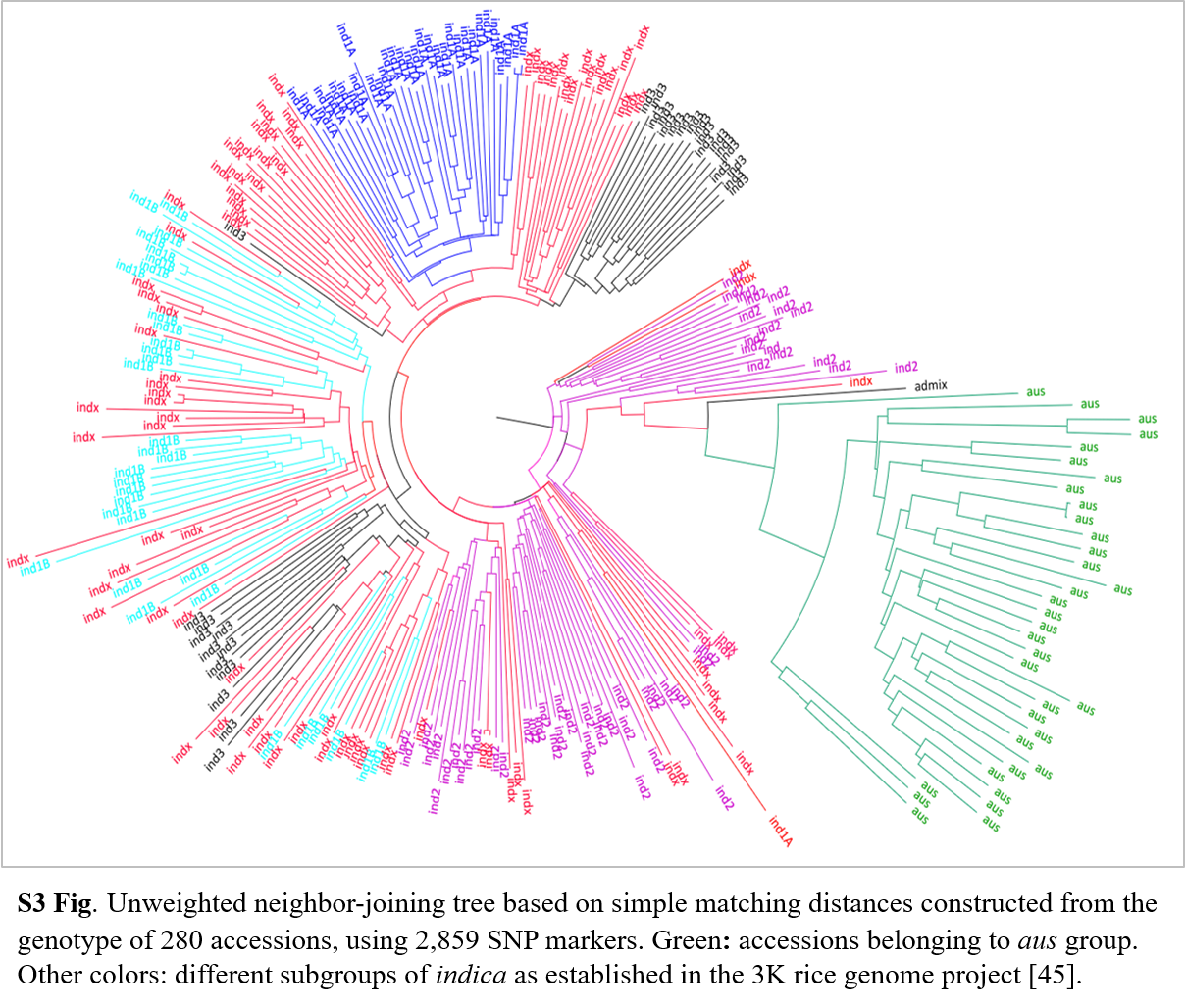

Supplement: S3 Fig — Green: accessions belonging to aus group. Other colors: different subgroups of indica as established in the 3K rice genome project [45]. (TIF) [file pone.0208871.s009.tif]
